# 

**Published:** 1875-06

**Authors:** 


CONTENTS.

| | PAGE |
|---|---|
| A Vegetable Diet | 165 |
| Mistakes Matrimonial | 167 |
| Coffee vs. Gout | 167 |
| Adhesive Plaster | 167 |
| Use of Silence | 168 |
| Flowers | 168 |
| The Toil of Shop-Girls | 169 |
| Mothers and Nurses | 169 |
| Rich Without Money | 169 |
| Good Natured People | 170 |
| Young Women | 171 |
| Longevity | 171 |
| Tobacco | 172 |
| Borrowing Trouble | 172 |
| Cooks that Kill | 173 |
| A Better Way | 174 |
| How Tobacco Hurts | 175 |
| Don't Try to do Too Many Things | 176 |
| Clean Cellars | 177 |
| Ventilating Sewers | 178 |
| Overworked School-Girls | 179 |
| Oatmeal | 180 |
| Our Summer at Maplewood. Chap. V. Griddle-Cakes and Other Things | 181 |
| A Fortune for Barbers | 186 |
| Women as Barbers | 187 |
| Unfermented Wine | 188 |
| Cremation | 188 |
| The House of Steinway | 189 |
| Calla Lilies | 190 |
| Trichiniasis | 191 |
| Tooth Tablets | 192 |
| An Act of Humanity | 193 |
| Infantile Diseases | 193 |
| Bad Breath | 194 |
| Heliotype | 195 |
| Home Adornment | 196 |

